# Encapsulation in Amylose Inclusion Complex Enhances the Stability and Release of Vitamin D

**DOI:** 10.3390/nu15051111

**Published:** 2023-02-23

**Authors:** Simiao Liu, Lingyan Kong, Tianzhuo Huang, Xiaohui Wei, Libo Tan, Hailing Luo, Hao Zhang

**Affiliations:** 1 College of Food Science and Nutritional Engineering, China Agricultural University, Beijing 100083, China; 2 Department of Human Nutrition and Hospitality Management, The University of Alabama, Tuscaloosa, AL 35487, USA; 3 State Key Laboratory of Animal Nutrition, College of Animal Science and Technology, China Agricultural University, Beijing 100193, China; 4 Beijing Laboratory of Food Quality and Safety, Department of Nutrition and Health, China Agricultural University, Beijing 100091, China; 5 Food Laboratory of Zhongyuan, Luohe 462300, China

**Keywords:** amylose, inclusion complex, encapsulation, vitamin D, stabilization, in vitro digestion

## Abstract

Vitamin D plays a significant role in the physiological functions of the human body. However, the application of vitamin D in functional foods is limited due to its sensitivity to light and oxygen. Therefore, in this study, we developed an effective method to protect vitamin D by encapsulating it in amylose. In detail, vitamin D was encapsulated by amylose inclusion complex, followed by structural characterization and evaluation of its stability and release properties. The results of X−ray diffraction, differential scanning calorimetry, and Fourier transform infrared spectroscopy showed that vitamin D was successfully encapsulated in the amylose inclusion complex, and the loading capacity was 1.96% ± 0.02%. The photostability and thermal stability of vitamin D after encapsulation was increased by 59% and 28%, respectively. In addition, in vitro simulated digestion showed that vitamin D was protected through the simulated gastric environment and can be released gradually in the simulated intestinal fluid, implying its improved bioaccessibility. Our findings provide a practical strategy for the development of functional foods based on vitamin D.

## 1. Introduction

Vitamin D is a fat−soluble vitamin possessing steroid−like effects in the body. The human body obtains vitamin D from two main sources: diet and ultraviolet (UV) irradiation from sunlight. Very few foods are naturally rich in or fortified with vitamin D. Thus, only less than 10% comes from food, and about 90% of vitamin D is synthesized in the skin upon exposure to UV irradiation. It is important to ensure adequate vitamin D intake by spending regular time in the sun. Ergocalciferol (vitamin D_2_) and cholecalciferol (vitamin D_3_) are the two vitamins that make up vitamin D. Both vitamin D_2_ and vitamin D_3_ are metabolized through a two−step hydroxylation process in the liver and kidneys, creating 25−hydroxyvitamin D and, subsequently, 1,25−dihydroxyvitamin D [1].

Vitamin D is necessary for immunological function and glucose metabolism, in addition to being crucial for calcium homeostasis and bone metabolism. Numerous diseases, like cancer, diabetes, cardiovascular disease (CVD), and multiple sclerosis, are at a higher risk when there is a low vitamin D level [2,3,4]. Calcium absorption needs vitamin D to be as effective as possible. Only 10 to 15% of the calcium in food may be absorbed without it. However, when vitamin D levels are adequate, calcium absorption increases by up to 40% [5,6]. It is also important to be mindful of vitamin D overdose, as it can lead to hypercalcemia and serious health risks such as dehydration, nausea, pain, polyuria, nephrocalcinosis, appetite loss, and renal failure. Even though vitamin D poisoning is incredibly uncommon, it can happen at overly high doses. 2000 IU per day is the maximum amount of vitamin D that may be consumed when taking supplements, but it is necessary to note that this does not guarantee zero risk of adverse effects, as the highest safe daily intake is not known [7].

Vitamin D deficiency due to insufficient sunlight exposure and unbalanced dietary intake has become a global health problem, affecting more than 1 billion children and adults around the world [8]. As people become more aware of UV protection, the risk of vitamin D deficiency increases significantly. The body’s capacity to produce vitamin D in the skin can be decreased by more than 95% by using sunscreen with an SPF of 30 [9]. Furthermore, factors including age, body mass index, living district, race, and clothing selection might impact vitamin D synthesis.

The most efficient strategy to raise vitamin D levels is through diet, as UV irradiation increases the risk of skin cancer and varies by region and season. However, dietary sources of vitamin D are restricted to a few foods, e.g., fatty fish, liver, fortified cereal, juice, and dairy, the accessibility of which is limited in many parts of the world [10,11]. Furthermore, the photosensitivity and thermal sensitivity of vitamin D further reduce its effectiveness and utilization in food production and processing [12,13]. For instance, Tsai et al. reported that after UV irradiation for 8 h, the endothermic peak at 82 °C of vitamin D gradually disappeared, indicating the degradation of vitamin D [14]. The degradation of vitamin D depends on the heating methods, the exposure time and the type of food. Previous research has investigated the effect of heat treatment and light on the vitamin D content of food. Heating canola oil at 180 °C for 30 min resulted in up to a 67% reduction in vitamin D_3_ [15]. The vitamin D_3_ concentration in fish showed a significant decrease after 6 h of sunlight exposure [16].

Encapsulation is an effective way to enhance the stability and regulate the release of the active components. In this technique, some materials coat the sensitive substances to avoid their loss. For example, Diarrassouba et al. discovered that the bioavailability of vitamin D and its resistance to gastrointestinal stimulation were both enhanced by microencapsulation through β−lactoglobulin−lysozyme [17]. David et al. reported that vitamin D was protected by nanocomplexation from thermal loss during pasteurization and from storage loss during simulated shelf−life tests [18]. Jannasari et al. showed that using gelatin and cress seed mucilage to encapsulate vitamin D could significantly increase its thermal resistance [19]. Despite several previous efforts in developing encapsulation systems for vitamin D, finding a low−cost wall material capable of improving its stability during storage, as well as regulating its release at its absorption site, is still not achieved.

Amylose, a linear polymer made of 1,4−linked glucose units, is one of the main components of starch. Based on the left−handed single helical structure it formed, amylose can encapsulate some small molecules, known as amylose–guest inclusion complex (IC). Since the inside chamber of this structure is hydrophobic and the outside surface of the helices is hydrophilic, it could be the perfect carrier for hydrophobic vitamin D. The guest compound can also be protected by the amylose IC from oxidation and strong acid, and the release of guest can be triggered as amylose starts to be digested in the small intestine [20,21].

Therefore, we developed and evaluated a novel encapsulation composition for vitamin D based on amylose–guest IC in this study. Complementary methods, including X−ray diffraction (XRD), differential scanning calorimetry (DSC), and Fourier transform infrared spectroscopy (FTIR), were used to characterize the prepared IC. Storage stability at 37 °C and upon UV irradiation was examined, and the release behavior of encapsulating vitamin D was examined via in vitro simulated digestion.

## 2. Materials and Methods

### 2.1. Materials

Vitamin D_3_ (Catalog No. 67970), amylose from potato (Catalog No. A0512), α−amylase from porcine pancreas (Type VI−B; ≥5 U/mg solid; Catalog No. A3176), pepsin (≥250 U/mg solid; Catalog No. P7000), and pancreatin from porcine pancreas (8 × USP specifications; Catalog No. P7545) were purchased from Sigma−Aldrich (Shanghai, China) Trading Co., Ltd. (Shanghai, China). Dipotassium hydrogen phosphate (K_2_HPO_4_) and monopotassium phosphate (KH_2_PO_4_) were obtained from Shanghai Yuanye Bio−Technology Co., Ltd. (Shanghai, China). Sodium chloride (NaCl) and sodium hydroxide (NaOH) were from Heowns Chemical Reagent Co., Ltd. (Tianjin, China). N−hexane, dimethyl sulfoxide (DMSO), methanol, acetonitrile, and other reagents were from Tianjin Guangfu Technology Development Co., Ltd. (Tianjin, China).

### 2.2. Preparation of Inclusion Complexes

An amount of 500 mg of amylose was mixed with 10 mL of 95% (*v*/*v*) DMSO for 30 min at 90 °C in each batch to dissolve it. Using a vortex mixer, the amylose dispersion was blended with 50 mg of pre−dissolved vitamin D in 1 mL of 95% (*v*/*v*) DMSO. After 30 min at 90 °C, the mixture was diluted with 25 mL of water and stored for an additional 15 min. The sample was centrifuged at 3000× *g* for 10 min after cooling for 24 h at 20 °C. The wet pellet was subsequently rinsed three times with a 50% (*v*/*v*) ethanol solution. Before being dried in a desiccator at 20 °C, the resulting precipitate was transferred to aluminum dishes with a tiny amount of ethanol. For further investigation, dry materials were ground into a fine powder.

### 2.3. Determination of Loading Capacity

The loading capacity of amylose–vitamin D IC was determined by enzymatic hydrolysis [21]. The encapsulation sample (20 mg) was mixed in 10 mg/mL of α−amylase phosphate solution (20 mM KH_2_PO_4_ and 20 mM K_2_HPO_4_ mixed in a 1:2 volume ratio) and placed in a water bath oscillator (150 rpm) at 37 °C for 4 h. After adding n−hexane to extract for 4 min and centrifuging at 3000× *g* for 4 min, the sample was filtrated with 0.22 μm nylon syringe. Vitamin D content in all samples was quantified by an HPLC instrument (Nexera UHPLC/HPLC, SHIMADZU, Kyoto, Japan) with UV detection at 264 nm and Waters Spherisorb^®^ C18 column (5 μm, 4.6 × 250 mm, Ireland), using the method reported earlier with few changes [22]. The injection volume was 20 μL, and the mobile phase was methanol/acetonitrile (20/80, *v*/*v*) at a flow rate of 1.3 mL/min. A series of concentrations ranging from 0 to 0.06 mg/mL were used to produce the standard curve for vitamin D standard solutions. The following equation was used to calculate the loading capacity:

(1) $$\mathrm{Loading}\;\mathrm{capacity}\;(\%)=\frac{\mathrm{weight}\;\mathrm{of}\;\mathrm{vitamin}\;D}{\mathrm{weight}\;\mathrm{of}\;\mathrm{sample}}\;\text{×}\;100$$

### 2.4. X−ray Diffraction (XRD) Analysis

An X−ray diffractometer (Philips X’PERT MPD, Malvern Panalytical Inc., Westborough, MA, USA) was used to obtain wide−angle XRD patterns. The ground samples were exposed to the radiation of Co Kα (0.179 nm) on the XRD machine. Using the HighScore software (Malvern Panalytical Inc., Westborough, MA, USA), data were converted to Cu Kα radiation−based 2θ values between 2θ = 5° and 30°.

### 2.5. Differential Scanning Calorimetry (DSC) Analysis

The samples’ thermal properties were examined using an autosampler−equipped DSC (DSC−60, SHIMADZU, Kyoto, Japan). Each sample, weighing about 4 mg, was placed in a DSC crucible made of an aluminum alloy, and deionized water was added to create a 10% (*w*/*v*) dispersion. The crucible was hermetically sealed and equilibrated for at least 3 h at room temperature (20 °C). Then, the sample was heated from 30 to 250 °C at a heating rate of 10 °C/min.

### 2.6. Fourier Transform Infrared Spectroscopy (FTIR) Analysis

A FTIR spectrometer (Spectrum 100−Spectrum, PerkinElmer Inc., Waltham, MA, USA) fitted with an attenuated total reflectance (ATR) accessory was used to collect the FTIR spectra of the samples. The recorded spectra were averaged for three scans in the wavelength region of 500 to 4000 cm^−1^ to obtain each spectrum, and the structural characterization of the embedded material was conducted. The OMNIC FTIR software 9 (Thermo Fisher Scientific Inc., Waltham, MA, USA) was used to collect the spectra.

### 2.7. Stability Tests

#### 2.7.1. Photostability

The photostability of the amylose–vitamin D IC was evaluated by comparing it with pure vitamin D and amylose–vitamin D physical mixture (PM). The samples of vitamin D, amylose–vitamin D PM, and amylose–vitamin D IC (about 200 mg each) were weighed into three plates and exposed to UV illumination (365 nm) for 12 h in the dark at room temperature (20 °C). After 2, 4, 6, and 12 h, samples were taken to extract and determine the retention of vitamin D according to the procedure described in Section 2.3. Each sample’s vitamin D content without UV exposure was regarded as 100% vitamin D retention [23].

#### 2.7.2. Thermal Stability

The thermal stability of the amylose–vitamin D IC was evaluated by comparing it with pure vitamin D and amylose–vitamin D physical mixture (PM). The samples of vitamin D, amylose–vitamin D PM, and amylose–vitamin D IC (about 200 mg each) were weighed into three plates and stored for 6 d in an incubator at 37 °C. After incubating for 1, 2, 4, and 6 d, the retention of vitamin D was determined following the same procedure as described above. Each sample’s vitamin D content without heat treatment was regarded as 100% retention of vitamin D.

### 2.8. In Vitro Release of Vitamin D

The same amount of vitamin D, amylose–vitamin D PM, and amylose–vitamin D IC was suspended in 50 mL of simulated gastric fluid (SGF, 1 L distilled water containing: NaCl, 2.0 g; Pepsin 0.6 g; 36% HCl, 7.0 mL; pH = 1.2), and then the suspension was placed in an oscillator (150 rpm) at 37 °C [24]. Following a 120 min digestion in SGF, digestion in simulated intestinal fluid (SIF) was performed: 10 mL SGF suspension was collected, and then added to the same amount of SIF (KH_2_PO_4_, 6.8 g/L; pig bile salt, 3.0 g/L; pancreatin, 1.0 g/L; and NaOH, 0.616 g/L; pH = 7.5). During the in vitro digestion, aliquots of samples were obtained at 0, 20, 40, 60 and 120 min of digestion from SGF and SIF, respectively. The samples were quickly placed in an ice bath for 2 min, and added n−hexane to extract vitamin D. To collect the supernatant immediately, the samples were centrifuged for 8 min at 6800× *g* and 4 °C. The released amount of vitamin D was determined according to the procedure described in Section 2.3. The following equation was used to calculate the in vitro release ratio of vitamin D:

(2) $$\mathrm{Vitamin}\;D\;\mathrm{release}\;(\%)=\frac{\mathrm{Amount}\;\mathrm{of}\;\mathrm{vitamin}\;D\;\mathrm{released}\;\mathrm{from}\;\mathrm{sample}}{\mathrm{Amount}\;\mathrm{of}\;\mathrm{vitamin}\;D\;\mathrm{initially}\;\mathrm{encapsulated}}\;\text{×}\;100$$

### 2.9. Statistical Analysis

The results are expressed as mean ± standard deviation (SD), and the number of samples was 3 per group (*n* = 3). Statistical analysis was tested in SPSS 26.0 (IBM Co., Armonk, NY, USA). The significant difference between different groups at each time point was determined by one−way analysis of variance (ANOVA) using Tukey multiple comparison tests. A value of *p* < 0.05 was considered statistically significant. Graphs were drawn by GraphPad Prism 9 (GraphPad Software, San Diego, CA, USA).

## 3. Results and Discussion

### 3.1. Characterization of the Inclusion Complex (IC)

#### 3.1.1. Loading Capacity

The IC sample obtained in this study was in a white powder form. The loading capacity was determined to be 1.96% ± 0.02% (*w*/*w*), revealing that the content of vitamin D in the encapsulation was 784 IU/mg. According to the Institute of Medicine of the National Academy of Sciences in the United States, the Recommended Dietary Allowance of vitamin D for adults is 600 IU per day and is 800 IU per day for those who are over 70 [25]. If the amylose–vitamin D IC were to be used for food fortification, it would only require the use of a very small amount (~1 mg) for daily food consumption, implying its easy utilization and minimum alteration to properties of the food matrix.

#### 3.1.2. X−ray Diffraction (XRD) Analysis

**Figure 1:**
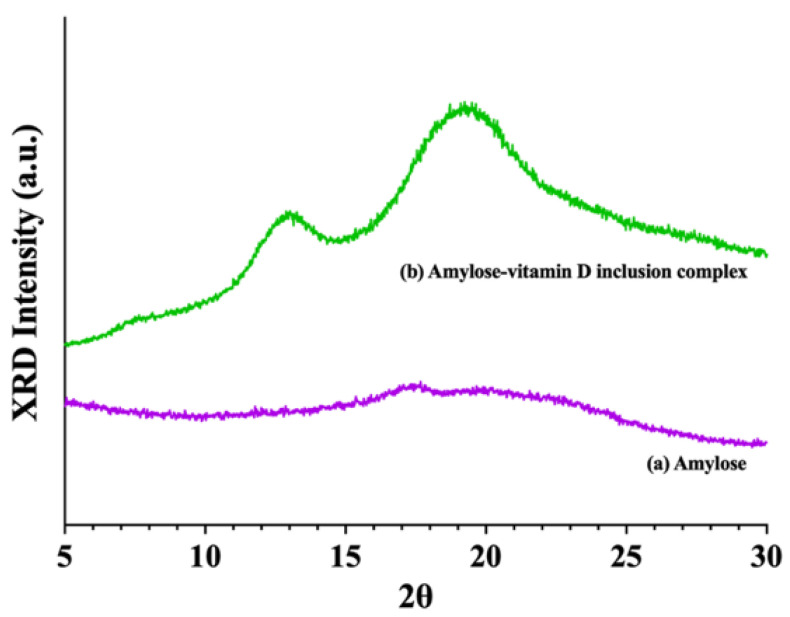
X−ray diffraction (XRD) patterns. (**a**) Amylose; (**b**) amylose–vitamin D inclusion complex (IC).

Figure 1 showed the XRD patterns of amylose and amylose–vitamin D IC. As expected, the amylose was largely amorphous and only showed a very weak peak at 17.5°, which was a characteristic peak of B−type starch [26,27]. Amylose molecules form left−handed single helices in the V−type structure, which has a central cavity of various sizes that can accommodate guest molecules. The V_6_, V_7_, and V_8_, which means each helical turn has 6, 7, and 8 glucose units, respectively, are the most well−characterized subtypes with different major diffraction peaks. The XRD pattern of the amylose–vitamin D IC sample showed peaks at 7.8°, 12.9°, and 19.3°, indicating the formation of the V−type pseudo−hexagonal orthorhombic crystals [28]. The strong diffraction peaks at 2θ = 7.8° and 2θ = 12.9° were associated with the structures of V_6a_ and V_6h_. The major diffraction peak at 2θ = 19.3° was between those characteristics of V_6h_ and V_7_, respectively. It implied a fractional helical turn between V_6_ and V_7_ or a mix of V_6_ and V_7_ helices. It should be noted that during conversions between the V types, the helices did not get larger or smaller. With such conversion, other conformational changes occurred [29]. In both cases, it could interrupt the arrangement of helices, and thus the sharpness of peaks and the degree of crystallinity. The existence of a V−crystalline structure indicated that vitamin D could be molecularly encapsulated [21].

#### 3.1.3. Thermal Analysis

**Figure 2:**
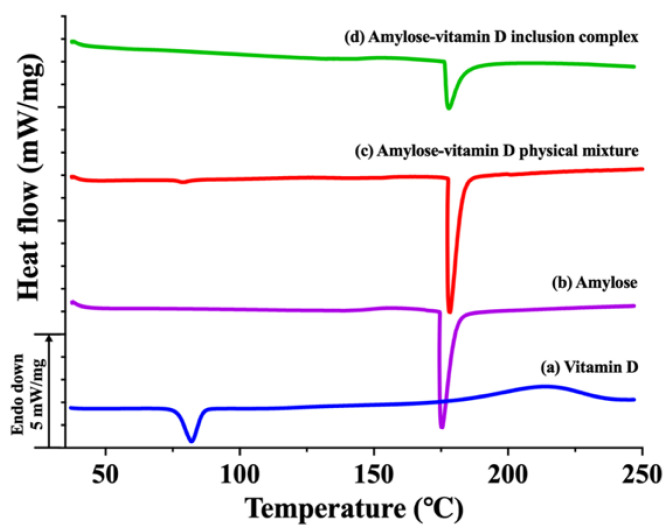
Differential scanning calorimetry (DSC) thermograms. (**a**) Vitamin D; (**b**) amylose; (**c**) amylose–vitamin D physical mixture (PM); (**d**) amylose–vitamin D inclusion complex (IC).

The DSC thermograms of vitamin D, amylose, amylose–vitamin D PM, and amylose–vitamin D IC are shown in Figure 2. At 82.1 °C, vitamin D showed a strong peak. Since vitamin D was insoluble in water, this endotherm was believed to be the melting of vitamin D instead of its dissolution [30]. The raw amylose showed a sharp peak at 175.9 °C, which also appeared in amylose–vitamin D PM and IC. This endotherm may be due to the degradation of amylose or the melting of certain higher order structure formed by amylose [31]. The physical mixture showed two endothermic peaks that corresponded to the melting point of vitamin D and amylose, indicating that there were no substantial covalent or noncovalent interactions between the guest molecules and amylose in the amylose–vitamin D PM. The low intensity of the peak of vitamin D was caused by the guest molecules’ low weight and the masking by the heat flow of amylose. However, the peak corresponding to vitamin D was not found in the IC sample, suggesting that vitamin D molecules were encapsulated in the amylose. The encapsulation of vitamin D in amylose can enhance its thermal resistance during heat processing. Combined XRD and DSC evidence suggested that vitamin D had been encapsulated in the helical cavity of amylose.

#### 3.1.4. Fourier Transform Infrared (FTIR) Investigation

**Figure 3:**
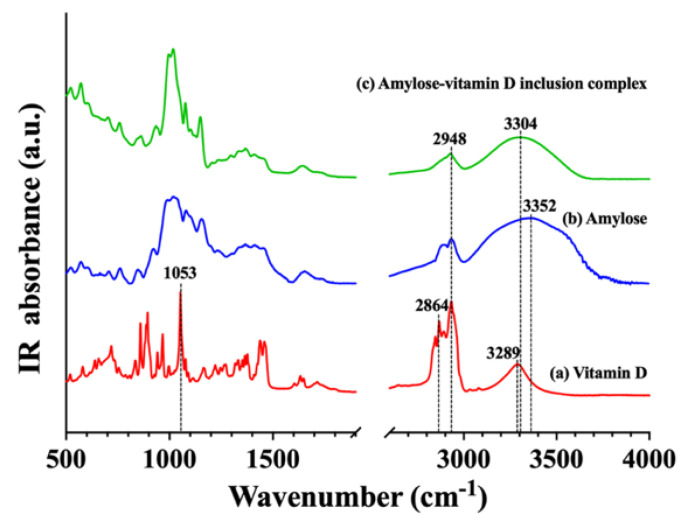
Fourier transform infrared spectroscopy (FTIR) spectra. (**a**) Vitamin D; (**b**) amylose; (**c**) amylose−vitamin D inclusion complex (IC).

The inclusion of the guest compound, vitamin D, in the samples was qualitatively identified by FTIR spectroscopy (Figure 3). O−H stretch (3289 cm^−1^), alkyl C−H stretch (2948 and 2864 cm^−1^), and an additional band showing C−H bend seen at 1053 cm^−1^ were identified on the spectra of vitamin D [14]. On the amylose spectrum, a strong absorption peak for amylose appeared at 3352 cm^−1^, which was due to amylose’s O−H stretching vibrations, and its width revealed the strength of the inter− and intra−molecular hydrogen interactions. The bands at 1366 cm^−1^ and 2948 cm^−1^ showed the angular deformation of C−H. The bands at 1152 cm^−1^ and 1077 cm^−1^ were associated with the stretching or bending vibrations of C−O bond and C−C bond [32]. The peak at 995 cm^−1^ was related to the C−O−C of α−1,4−glycosidic linkages [33]. On the amylose–vitamin D IC spectrum, the major absorption peaks of amylose were still observed, but the skeletal vibrations of vitamin D (2864 cm^−1^ and 1053 cm^−1^) vanished, which convincingly showed encapsulation of the vitamin D in amylose. Moreover, vitamin D loading caused the peak at 3352 cm^−1^ to move to 3304 cm^−1^, indicating that vitamin D and amylose formed a hydrogen bond. Amylose and vitamin D molecules’ OH groups may be crucial in the creation of hydrogen bonding that caused the spectra to change following encapsulation.

### 3.2. Stability Studies

#### 3.2.1. Photostability

**Figure 4:**
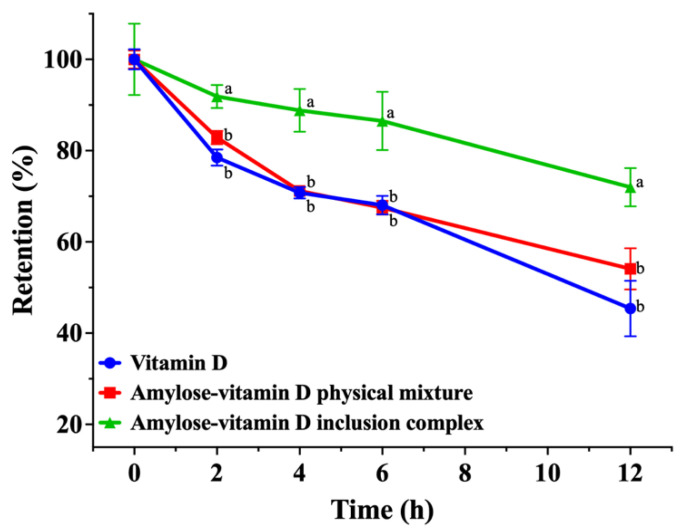
Photostability of vitamin D, amylose–vitamin D physical mixture (PM), and amylose–vitamin D inclusion complexes (IC). The results are expressed as mean ± standard deviation for three measurements (*n* = 3). The different letters indicate significant differences at each time point, *p* < 0.05.

UV irradiation is a form of electromagnetic radiation that has a wavelength between visible light and X−rays. Together with the effects of oxygen and moisture, it may induce the photodegradation of numerous substances even though it is not ionizing radiation. Therefore, UV irradiation is a great way to evaluate photostability by measuring the rate of degradation for photolabile compounds. Figure 4 showed the retention of vitamin D in samples subject to UV irradiation. With prolonged UV exposure, the retention of vitamin D in pure vitamin D sample and amylose–vitamin D PM significantly decreased. After 2 h of direct UV irradiation, the retention of vitamin D in amylose–vitamin D IC group started to show significant differences (*p* < 0.05). Only 45.4% ± 5.0% and 54.1% ± 3.7% of vitamin D were retained in pure vitamin D and amylose–vitamin D PM after 12 h of illumination, respectively. For amylose–vitamin D IC samples, the retention of vitamin D was 72.0% ± 3.4% after 12 h of illumination and was significantly higher than the other groups (*p* < 0.05). The photostability of vitamin D is a major hurdle in vitamin D−enriched foods such as milk and infant formula. The amylose–vitamin D IC significantly enhanced the photostability of vitamin D, guaranteeing that it had a longer storage time. A physical barrier against light may be created for vitamin D encapsulated in amylose, providing new perspectives on the protection of photolabile compounds [34].

#### 3.2.2. Thermal Stability

**Figure 5:**
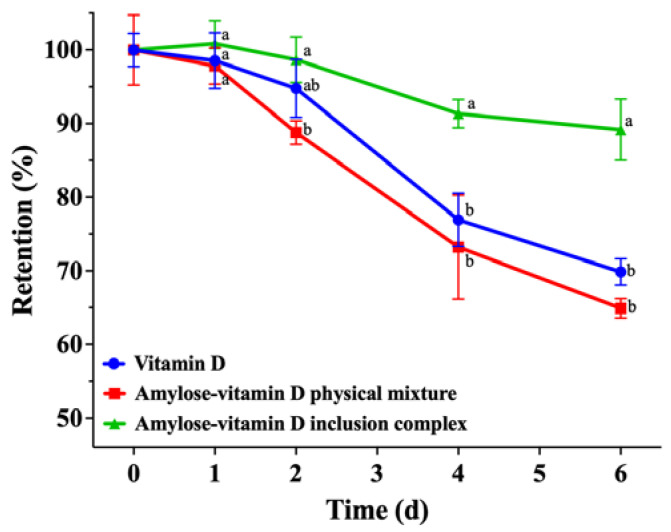
Thermal stability of vitamin D, amylose–vitamin D physical mixture (PM), and amylose–vitamin D inclusion complexes (IC). The results are expressed as mean ± standard deviation for three measurements (*n* = 3). The different letters indicate significant differences at each time point, *p* < 0.05.

In addition to blocking UV irradiation, encapsulating vitamin D into the polymeric matrix may also be a good strategy to offer defense against other harsh conditions. The results of the thermal stability experiments are shown in Figure 5. After incubating at 37 °C for 2 d, only about 1.3% of vitamin D was degraded in amylose–vitamin D IC group, which corresponded to a 98.7% ± 2.5% retention of vitamin D. After 4 d of storage at 37 °C, the effectiveness of IC in improving the thermal stability of vitamin D started to show, and its retention of vitamin D was significantly higher than vitamin D and amylose–vitamin D PM groups (*p* < 0.05). After the heat treatment for 6 d, the retention of vitamin D was 89.2% ± 3.4% in the amylose–vitamin D IC, compared with 69.9% ± 1.5% in the pure vitamin D and 64.9% ± 1.1% in the amylose–vitamin D PM sample. The dense crystalline structure of amylose–vitamin D IC precluded direct contact between vitamin D and oxidizing substances, therefore providing a protective effect on vitamin D [18].

### 3.3. In Vitro Release Analysis

**Figure 6:**
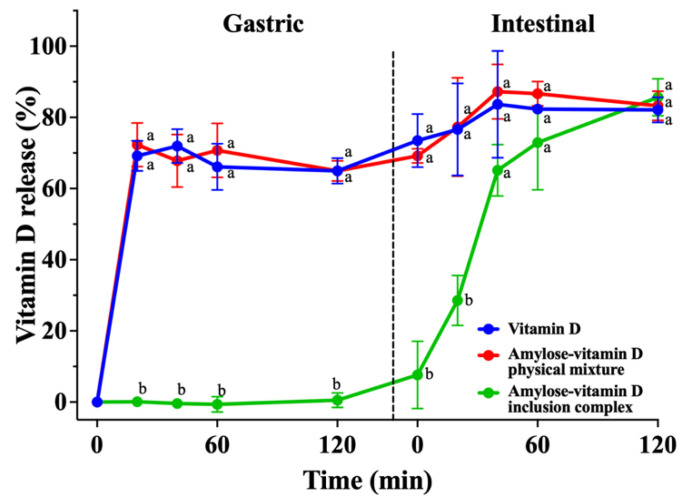
In vitro release profiles of vitamin D, amylose–vitamin D physical mixture (PM), and amylose–vitamin D inclusion complexes (IC). The results are expressed as mean ± standard deviation for three measurements (*n* = 3). The different letters indicate significant differences at each time point, *p* < 0.05.

The main purposes of encapsulation shall not only be to improve the stability of the guest compound, but also to facilitate or regulate the release of the guest compound for its absorption. By analyzing the content of vitamin D in SGF and SIF over a period of 240 min, the release rates of vitamin D in various groups are shown in Figure 6. Since vitamin D digestion and absorption generally occur in the small intestine, a large intestine simulation was not carried out. The in vitro release characteristics of vitamin D from the IC were assessed in comparison to both its pure form and mixture with amylose. The vitamin D and amylose–vitamin D PM groups showed substantial release before the first 20 min of the simulated gastric period, with release rates of 69.2% ± 3.5% and 72.3% ± 5.0%, respectively. In contrast, vitamin D was scarcely released from amylose–vitamin D IC, presumably due to the lack of amylase in the simulated gastric period, indicating that the inclusion was relatively stable after entering the simulated gastric fluid [35]. During the simulated intestinal digestion, the rate of vitamin D release remained relatively constant in the pure vitamin D and amylose–vitamin D PM groups for the first 40 min, the release rate of which reached 83.7% ± 12.2% and 87.2% ± 6.3%, respectively. Since the samples were exposed to digestive enzymes, the release of vitamin D from its IC was accelerated, reaching 65.1% ± 5.9% at 40 min. The IC continued to release vitamin D for the next 80 min but at a lower rate, reaching the same level of total release as the other groups at 120 min. The results showed that the amylose–vitamin D IC allowed better protection of vitamin D through the simulated acidic stomach environment and enabled slow and sustained release in simulated intestinal conditions, which could benefit the absorption of vitamin D. Considering the complex process of vitamin D digestion and absorption in the human body, further investigations using cell and animal models are required to test the bioavailability and the toxicity of inclusion complexes after ingestion.

## 4. Conclusions

In the present study, we successfully encapsulated vitamin D in amylose inclusion complex (IC) as evidenced by complementary XRD, DSC, and FTIR techniques. XRD patterns confirmed that amylose molecules formed left−handed single helices in the V−type structure to encapsulate vitamin D. DSC thermograms showed the encapsulation of vitamin D in amylose may increase its thermal resistance against heat processing. Based on FTIR spectroscopy, OH groups of vitamin D and amylose molecules may play an important role in the formation of hydrogen bonding. After 12 h of direct UV irradiation, the photostability of vitamin D was significantly improved in the encapsulated form. Additionally, the thermal stability of vitamin D showed a significant improvement after incubating at 37 °C for 6 d. In vitro digestion revealed that vitamin D was not released in the simulated gastric buffer but exhibited a gradual and sustained release behavior in the simulated intestinal fluid, which implies a potentially higher absorption and bioaccessibility of vitamin D in the encapsulated form. Such amylose–vitamin D IC has the potential to be utilized in a variety of food products.

## Data Availability

The data presented in this study are available on request from the corresponding author. The data are not publicly available to preserve scientific integrity of research methodology.
